# Is black tar heroin use associated with wound botulism? A report of two Hispanic patients

**DOI:** 10.1002/ccr3.1622

**Published:** 2018-05-29

**Authors:** Ihtesham A. Qureshi, Mohtashim A. Qureshi, Anantha‐Ramana Vellipuram, Darine Kassar

**Affiliations:** ^1^ Neurology Department Texas Tech University Health Sciences Center El Paso TX USA

**Keywords:** heroin abuse, paralysis, skin popping, trivalent equine antitoxin, wound botulism

## Abstract

Wound botulism is a potentially lethal condition that can cause paralysis. Its association with black tar heroin is a well‐established fact. It is essential to alert clinicians in recognizing the patients with history of injection drug abuse presenting with clinical features of botulism early on admission for prompt diagnosis and treatment.

We present 2 cases (48‐year‐old Hispanic Man & 34‐year‐old Hispanic Woman) both with past medical history of heroin abuse was brought to the emergency department with acute respiratory failure, proximal muscle weakness of upper and lower extremity, neck flexor muscle weakness, and diplopia. Urine drug was consistent with opioids. Multiple areas of visible skin popping, a technique of injecting black tar heroin into extravenous subcutaneous sites are seen on the right and left areas of thigh associated with the development of botulism^1^ (Figures 1 and 2). Both patients were immediately transferred to the medical intensive care unit and were placed on mechanical ventilation. There was a high index of suspicion for wound botulism and trivalent equine antitoxin was administered. One was discharged to a long‐term skilled nursing facility after tracheostomy, percutaneous endoscopic gastrostomy (PEG) tube placement, and the other patient was discharged home with full recovery. As wound botulism is a potentially lethal condition caused by clostridium botulinum (anaerobic gram‐positive bacterium, which produces a neurotoxin that causes paralysis), early recognition and prompt treatment can decrease the overall mortality, total length of hospital stay and thus reduces the financial burden on the health‐care systems.^2^

**Figure 1:**
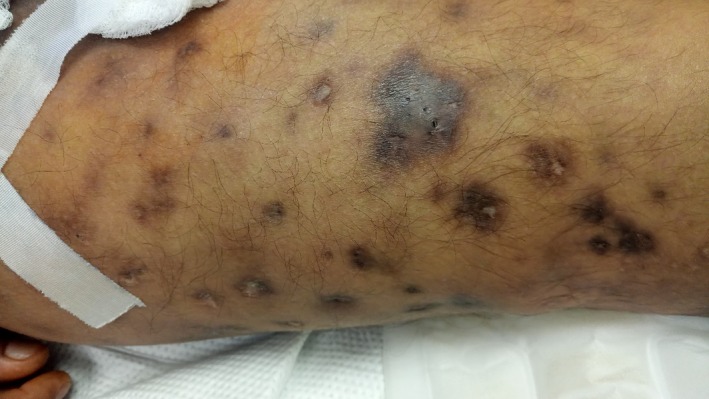
Picture depicting multiple areas of visible skin popping sites with abscesses at the site of black tar heroin administration in the right thigh associated with wound botulism

**Figure 2:**
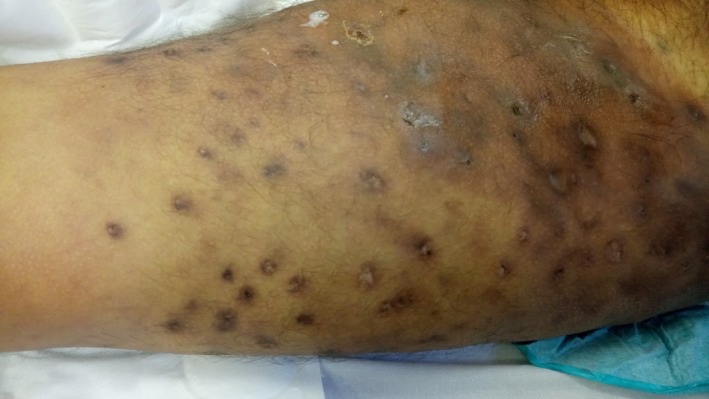
Picture depicting multiple areas of visible skin popping sites with abscesses at the sites of black tar heroin administration in the left thigh associated with wound botulism

## CONFLICT OF INTEREST

None declared.

## AUTHORSHIP

IAQ: involved in manuscript writing; MAQ: involved in critical revision of the manuscript; ARV and DK: involved in patient care.
